# Management of patients with high-risk and advanced prostate cancer in the Middle East: resource-stratified consensus recommendations

**DOI:** 10.1007/s00345-019-02872-x

**Published:** 2019-07-11

**Authors:** Deborah Mukherji, Bassem Youssef, Christelle Dagher, Albert El-Hajj, Rami Nasr, Fadi Geara, Danny Rabah, Saad Al Dousari, Rabih Said, Raja Ashou, Wassim Wazzan, Michel Jabbour, George Farha, Nibras Al Hamdani, Yousuf Al Hallaq, Hassan Ghazal, Haifa Dbouk, Bassel Bachir, Clement El Khoury, Ghazi Sakr, Hero K. Hussain, Khaled Sayyid, Khaled Ibrahim, Mohammad Haidar, Nicolas Zouain, Nizar Bitar, Walid Alameh, Fadi Abbas, Sami Faddoul, Elie Nemer, Georges Assaf, Fadi Farhat, Muhammad Bulbul, Sally Temraz, Ali Shamseddine, Silke Gillessen, Aurelius Omlin, Raja Khauli

**Affiliations:** 1 Division of Hematology/Oncology, Department of Internal Medicine, American University of Beirut Medical Center, Riad El Solh, 1107 2020 Beirut, Lebanon; 2 Department of Radiation Oncology, American University of Beirut Medical Center, Beirut, Lebanon; 3 Division of Urology, Department of Surgery, American University of Beirut Medical Center, Riad El Solh, 1107 2020 Beirut, Lebanon; 4 Department of Surgery, College of Medicine, King Saud University, Riyadh, Saudi Arabia; 5 Department of Surgery, Faculty of Medicine, Kuwait University, Kuwait City, Kuwait; 6 Division of Hematology/Oncology, Department of Internal Medicine, Saint Georges Hospital University Medical Center, Beirut, Lebanon; 7 Department of Radiology, Saint Georges Hospital University Medical Center, Beirut, Lebanon; 8 Division of Urology, Department of Surgery, Saint Georges Hospital University Medical Center, Beirut, Lebanon; 9 Department of Radiation Oncology, Mount Lebanon Hospital, Faculty of Medicine-University of Balamand, Beirut, Lebanon; 10 Department of Surgery, Medical City Teaching Hospital Baghdad, Baghdad, Iraq; 11 Division of Hematology/Oncology, Department of Internal Medicine, Clemenceau Medical Center, Beirut, Lebanon; 12 Division of Oncology, Jamal Amel Hospital, Tyre, Lebanon; 13 Department of Radiation Oncology, Hotel Dieu de France, Saint Joseph University Faculty of Medicine, Beirut, Lebanon; 14 Division of Urology, Department of Surgery, Mount Lebanon Hospital, Beirut, Lebanon; 15 Department of Radiology, American University of Beirut Medical Center, Beirut, Lebanon; 16 Department of Surgery, Makassed Hospital, Beirut, Lebanon; 17 Division of Hematology/Oncology, Department of Internal Medicine, Hammoud Hospital University Medical Center, Saida, Lebanon; 18 Department of Radiation Oncology, Clemenceau Medical Center, Beirut, Lebanon; 19 Division of Hematology/Oncology, Department of Internal Medicine, Sahel General Hospital, Beirut, Lebanon; 20 Department of Surgery, Sahel General Hospital, Beirut, Lebanon; 21 Middle East Tumor Institute, Beirut, Lebanon; 22 Doctors Center Radiology, Beirut, Lebanon; 23 Department of Urology, Saint Joseph University, Beirut, Lebanon; 24 Division of Cancer Sciences, University of Manchester and the Christie, Manchester, UK; 25 Department of Medical Oncology and Haematology, Cantonal Hospital Saint Gallen, University of Bern, Bern, Switzerland

**Keywords:** Prostate cancer, Middle East, Resource-stratified recommendations, Consensus, Multidisciplinary

## Abstract

**Purpose:**

Prostate cancer care in the Middle East is highly variable and access to specialist multidisciplinary management is limited. Academic tertiary referral centers offer cutting-edge diagnosis and treatment; however, in many parts of the region, patients are managed by non-specialists with limited resources. Due to many factors including lack of awareness and lack of prostate-specific antigen (PSA) screening, a high percentage of men present with locally advanced and metastatic prostate cancer at diagnosis. The aim of these recommendations is to assist clinicians in managing patients with different levels of access to diagnostic and treatment modalities.

**Methods:**

The first Advanced Prostate Cancer Consensus Conference (APCCC) satellite meeting for the Middle East was held in Beirut, Lebanon, November 2017. During this meeting a consortium of urologists, medical oncologists, radiation oncologist and imaging specialists practicing in Lebanon, Syria, Iraq, Kuwait and Saudi Arabia voted on a selection of consensus questions. An additional workshop to formulate resource-stratified consensus recommendations was held in March 2019.

**Results:**

Variations in practice based on available resources have been proposed to form resource-stratified recommendations for imaging at diagnosis, initial management of localized prostate cancer requiring therapy, treatment of castration-sensitive/naïve advanced prostate cancer and treatment of castration-resistant prostate cancer.

**Conclusion:**

This is the first regional consensus on prostate cancer management from the Middle East. The following recommendations will be useful to urologists and oncologists practicing in all areas with limited access to specialist multi-disciplinary teams, diagnostic modalities and treatment resources.

**Electronic supplementary material:**

The online version of this article (10.1007/s00345-019-02872-x) contains supplementary material, which is available to authorized users.

## Introduction

**Fig. 1:**
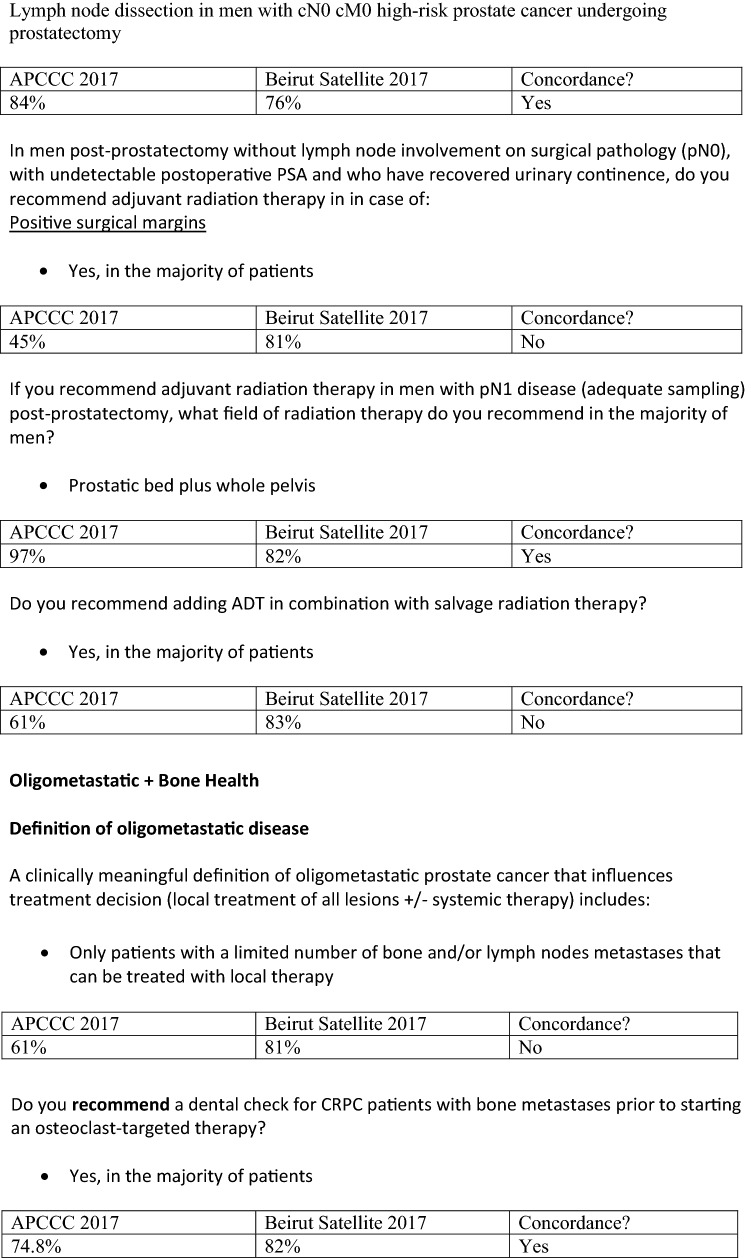

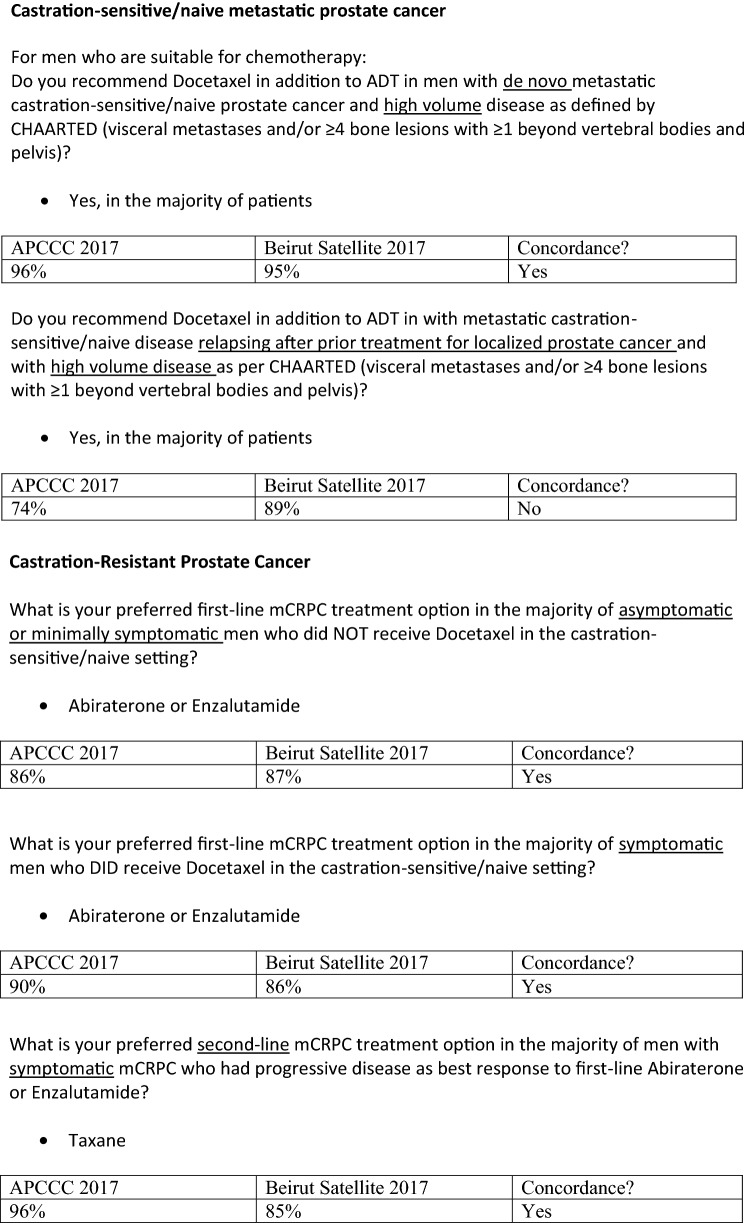

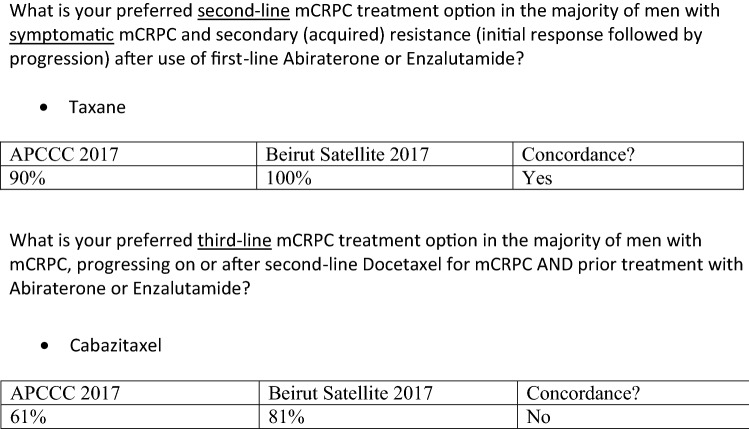
Areas of consensus (≥ 75% agreement) APCCC 2017 compared to Beirut Satellite Meeting 2017

The 2017 Advanced Prostate Cancer Consensus Conference (APCCC) was held in St Gallen, Switzerland in March 2017 during which a panel of 60 international experts voted on 150 questions addressing controversial topics in prostate cancer management [1]. The first APCCC Satellite Meeting for the Middle East was held in Beirut, Lebanon in November 2017 in conjunction with the Middle East Prostate Cancer Consortium (MEPCC) comprised of urologists, medical oncologists, radiation oncologists and imaging specialists largely from Lebanon, Syria and Iraq with expert urologists from Kuwait and Saudi Arabia joined by the co-chair of the APCCC Dr Aurelius Omlin from St Gallen. During this satellite meeting, faculty members presented brief clinical updates with a focus on topics of particular relevance to the Middle East region and all attendees were asked to vote on a selection of the APCCC questions (see supplemental material). Consensus was declared if ≥ 75% of participants who did not vote for “unqualified” or “abstain” and chose the same option [2] (see Fig. 1). The meeting was organized by the Continuing Medical Education office of the American University of Beirut and co-chaired by Dr Deborah Mukherji (medical oncologist) and Dr Raja Khauli (urologist and panel member for the APCCC 2017 meeting).

The manuscript published following the APCCC 2017 provides a guide for clinicians to assist in the discussions with patients as part of a multidisciplinary decision-making process particularly in areas lacking clear evidence from randomized clinical trials on which to base treatment recommendations [1]. Due to the rapid changes in prostate cancer management since this publication of the APCCC recommendations in 2017, an additional workshop was held in Beirut in March 2019 to update our resource-stratified consensus recommendations specifically for the Middle East using a modified-Delphi method. Resource-stratified recommendations are based on expert-opinion and structured in-line with a simplified version of the resource-stratification levels proposed by the Breast Health Global Initiative (BHGI) and adopted by the American Society of Clinical Oncology (ASCO) [3]. We have stratified our recommendations between two levels rather than four since the choice of treatment in the Middle East region is not just dependent on financial resources but also available expertise. We did not feel that for the needs of practitioners in the region, making finer discrimination between the four levels would be of added value since there would be considerable overlap and repetition. We emphasize the importance of referral to centers with appropriate expertise as needed wherever possible. Significant variations in coverage of health-care costs exist both within and between countries. Systems of governmental health-care coverage, private insurance and self-payment operate in parallel. In a region lacking access to specialist multidisciplinary care in many areas, these recommendations are not designed to replace evidence-based guidelines, however, may assist in management decisions for patients with different levels of access to diagnostic and treatment modalities.

## Epidemiology of prostate cancer in the Middle East

Prostate cancer is the second most commonly diagnosed cancer in men worldwide, with an incidence of 1,276,106 in 2018 and the 5th most common cause of cancer mortality worldwide with 358,989 fatalities in 2018, according to Globocan [4]. Australia, Europe and Northern America have the highest age-standardized incidence rate of prostate cancer in 2018, when Asia has the lowest incidence rate [4].

A dramatic increase in prostate cancer incidence has been globally identified in the past few decades since the introduction of PSA screening [5]. In 2018, prostate cancer has been identified as the most common cancer in men in all the continents except for Asia where it is the fifth most common cancer (Table 1) [4]. Nevertheless, it is worth noting that the reported incidence in the past 5 years in the US has dropped since the United States Preventative Services Task Force (USPSTF) issued a recommendation against screening in 2012, with concomitant rise in locally advanced disease and nodal metastasis at diagnosis [6].

**Table 1:** Incidence of Prostate cancer in 2018 worldwide

Area	Incidence of prostate cancer in 2018	Age-standardized ratio per 100,000
Europe	449,761	62.1
Asia	297,215	11.5
North America	234,278	73.7
Latin America and the Caribbean	190,385	56.4
Africa	80,971	26.6
Oceania	23,496	79.1

Partly due to the lack of awareness and regular screening in the Middle East, plus potential deficiencies in regional cancer registries, the incidence of prostate cancer is low compared to the rest of the world. Lebanon, a country with advanced tertiary care referral centers, has the highest rate of prostate cancer in the region due to high use of PSA screening with 39.3 per 100,000 in most recent data in 2018 (Table 2).

**Table 2:** Incidence of prostate cancer in the Middle East region, 2018

Middle East country	Incidence of prostate cancer in 2018	Age-standardized ratio per 100,000
Lebanon	1503	39.3
Iraq	556	6.6
Jordan	397	14.7
Kuwait	221	21.6
Oman	145	12.7
Qatar	73	15.5
Saudi Arabia	607	6.1
Syria	1136	20.1
Algeria	2578	13.0
Egypt	3109	9.5
Libya	317	15.6
Morocco	3990	22.7
Tunisia	819	12.3

## Diagnosis and management of high-risk localized and locally advanced prostate cancer

In common with many parts of the world, cancer care in the Middle East is highly variable with specialist multidisciplinary care limited to few academic medical centers in the region. PSA screening is not routinely practiced, and our panelists reported that a significant proportion of men with prostate cancer present with locally advanced and metastatic disease. Few prospective data are available, however, a single-institution study from the American University of Beirut, Lebanon showed that up to 25% of patients presented with locally advanced or metastatic disease including 16% with M1 disease [7].

### Imaging at diagnosis

Recent data from the PRECISION and PROMIS studies have established the value of performing magnetic resonance imaging (MRI) prior to prostate biopsy in men with suspected prostate cancer, however, our panel discussed the fact that few radiologists in the region have specific training in reading prostate MRI according to the prostate imaging reporting and data system (PIRADS) classification [8, 9]. For patients who have access to MRI pre-biopsy, targeted and random biopsies from the prostate are recommended, either “cognitive” targeting or image-guided according to the expertise of the treating team. For patients without access to MRI pre-biopsy, trans-rectal ultrasound (TRUS)-guided biopsy remains standard of care. If TRUS-guided biopsy is not available, patients should be referred to centers offering this modality; finger-directed biopsy of the prostate should only be performed in patients with grossly advanced local disease. For patients with high-risk disease at diagnosis, the panel considered that the current standard of care remains bone scan and cross-sectional imaging with either computed tomography (CT) or MRI. However, for patients with access to positron emission tomography (PET)-PSMA or whole-body MRI scans (so-called ‘next-generation imaging’), these imaging modalities are increasingly being utilized for staging, particularly PET-PSMA in Lebanon and Saudi Arabia (Table 3). To date there is no evidence that the use of next-generation imaging for staging improves outcomes. The panel discussed the controversy surrounding the management of patients found to have distant metastatic disease on next-generation imaging since treatment guidelines are based on patients staged with conventional modalities. We recommend that patients diagnosed with stage 4 disease on next-generation imaging should not be offered radical prostatectomy outside clinical trials, however, given recent data from the STAMPEDE trial, radiation to the primary in low-volume metastatic disease is now standard of care and should be offered [10].

**Table 3:** Resource-stratified recommendations for imaging at diagnosis of prostate cancer

Resource-level	Imaging prior to biopsy	Biopsy	Imaging to rule-out metastatic disease in high-risk patients
Basic/limited	None	TRUS-guided biopsy or finger directed in grossly advanced local disease	Bone scan plus or minus CT or MRI
Enhanced/maximal	MRI	Targeted plus random Cognitive or fusion-image guided if local expertise and access	Bone scan plus CT PET-PSMA if accessible Whole-body MRI if accessible and local expertise

### Initial management of localized/locally advanced disease

The definition of high-risk localized and locally advanced prostate cancer varies between guidelines and inclusion criteria for key clinical trials such as the STAMPEDE study [1]. For the APCCC 2017 St Gallen meeting, the European Association of Urology (EAU) guideline definition was used (high-risk localized disease: PSA level > 20 ng/ml, or Gleason score > 7 or > T2c; locally advanced disease: any PSA level, any Gleason score, cT3-4, or cN+).

In regions with limited access to primary care physicians, the majority of men with prostate cancer present to a general urologist. Due to lack of formal multidisciplinary tumor board meetings outside academic medical centers, the urologist who makes the diagnosis of prostate cancer is usually responsible for setting the treatment plan and making referrals to other specialists. Our panel recommends that ideally all men with locally advanced prostate cancer should be discussed with and seen by a radiation oncologist and a medical oncologist for formulation of the management plan (Table 4). Physicians with no access to an on-site tumor board can present cases virtually or in person at institutions where multidisciplinary meetings are being held. Other options include direct telephone/email contact between local urologists, medical oncologists and radiation oncologists. Multidisciplinary discussion is essential at diagnosis but also important at relapse following radical local therapy.

**Table 4:** Resource-stratified recommendations for initial management of localized prostate cancer requiring therapy

Resource-level	Multidisciplinary discussion	Surgical management of localized prostate cancer	Radiation for localized prostate cancer
Basic/limited	Review of published guidelines Discussion of options with patient by primary physician Telephone/email discussion between urologist/oncologist/radiation oncologist Patient referral to other specialists for management	Open radical prostatectomy ± pelvic lymph node dissection (for non-low risk patients)	External-beam radiation therapy with addition of ADT for intermediate-high risk disease
Enhanced/maximal	Face-to-face tumor board with imaging and pathology review Patient referral to urologist, oncologist and radiation oncologist to discuss options for management	Consider radical prostatectomy (open or robotic-assisted laparoscopic depending on local expertise) Extended lymph node dissection with for men with high-risk disease	Consider IMRT with ADT for intermediate/high-risk disease ± moderate hypofractionation Consider brachytherapy if available and appropriate

The St Gallen APCCC 2017 conference did not address the choice of primary treatment for high-risk localized and locally advanced prostate cancer. In the Middle East region since many patients are diagnosed with prostate cancer by general urologists, our panelists noted that in some cases men with locally advanced or metastatic disease at diagnosis are offered androgen-deprivation therapy (ADT) alone and not referred for radiation or additional systemic therapy despite new data suggesting a survival benefit to treatment intensification for this group of patients [11–14]. Our panelists also discussed that at the other end of the spectrum, patients presenting with locally advanced disease may be offered surgery as part of a multimodality treatment; patients have to be informed about the high possibility of requiring adjuvant radiation therapy. Our group is working on the development of patient information in regional languages describing different treatment modalities and their potential toxicities.

### Surgical management of high-risk localized and locally advanced prostate cancer plus or minus adjuvant radiation therapy

Our panel discussed the fact that younger patients without medical co-morbidities are increasingly opting for primary surgery; however, education about the possible need for adjuvant radiation therapy should be discussed with all patients, ideally with referral to a radiation oncologist (Table 4). Our panelists agreed with the APCCC 2017 consensus regarding pelvic lymph node (PLND) dissection for the majority of men with high-risk prostate cancer undergoing radical prostatectomy (Fig. 1). Extended PLND dissection to the level of the common iliac arteries was also a consensus recommendation, however, our panel qualified that this should only be undertaken by surgeons with appropriate training and expertise.

For centers with appropriate resources and expertise, open radical prostatectomy and robotic-assisted laparoscopic prostatectomy are options to discuss with patients. Ideally, patients with high-risk and locally advanced disease requesting surgery should be referred to high-volume centers. Our panel stressed the importance of patient education regarding rigorous follow-up post-prostatectomy using PSA monitoring to ensure that patients who will require salvage radiation therapy will be identified at an appropriately early time period with low PSA levels (see salvage radiation section).

### Adjuvant radiation after radical prostatectomy

Patients post-prostatectomy with an undetectable post-operative PSA but high-risk pathologic features should be considered for adjuvant radiation therapy; however, the decision between offering adjuvant radiation versus early salvage radiation is controversial and an overall survival benefit associated with pure adjuvant treatment has not been demonstrated [15]. The results of the on-going RADICALS (NCT00541047) and RAVES (NCT00860652) studies which have been designed to compare adjuvant and early savage radiation therapy may inform future practice.

Our panel reached consensus that the majority of high-risk patients with positive surgical margins should be offered adjuvant radiation therapy. The importance of giving adjuvant therapy based on positive margins alone has been debated in the literature; however, recent data suggest that the risk of recurrence and even prostate cancer-specific mortality may be higher in patients with positive margins and high-risk disease [16]. For patients with seminal vesicle involvement or Gleason grade group 5 tumors, or even certain patients with node-positive tumors but no other high-risk features and undetectable post-operative PSA, the option of adjuvant versus early salvage radiation can be discussed. Adjuvant treatment for node-negative disease is generally given to the prostate bed and pelvis with ADT for 6 months [17]; however, consensus was not reached in our panel on this issue, as results from the NRG/RTOG 0534 trial are still not fully published. Our panel reached consensus that adjuvant radiation for men with node-positive disease should be given to the prostate bed plus whole pelvis with at least 18–36 months of ADT.

The results of randomized studies comparing purely adjuvant versus salvage radiation therapy are awaited, however, in terms of cost-effectiveness in low-resource settings, if appropriate PSA monitoring can be undertaken our panel recommends deferring radiation to the early salvage setting in men with evidence of rising PSA post-prostatectomy.

### Salvage radiation after radical prostatectomy

Men who fail to achieve an undetectable PSA (< 0.2 ng/ml) post-prostatectomy [18] and those with rising PSA after an initial drop to undetectable levels are candidates for salvage radiation. Current data suggest that starting early salvage radiation therapy before the PSA rises to 0.5 will have better outcomes [19, 20]. Our panel reflected that many patients do not have appropriate follow-up of post-prostatectomy and present for salvage with much higher PSA levels. On the other extreme, patients may opt to measure their PSA post-operatively at very frequent intervals and present with evidence of rising PSA at lower levels than the standard 0.2 ng/ml definition of biochemical recurrence. The panel considered that recommended salvage radiation in men with a confirmed rising PSA prior to the 0.2 ng/ml threshold is appropriate (two successive rises of PSA) [21].

The panel discussed that if resources are available, a PET-PSMA can be useful in the context of patients presenting post-prostatectomy with PSA > 0.5 ngml to evaluate for nodal or distant metastatic disease [22] and tailor the need and extent of radiation salvage.

A consensus was reached regarding the recommendation of ADT (LHRH-Agonist preferred over anti-androgen) with salvage radiation therapy; however, there was no consensus over the duration and in general 6–12 months are recommended according to the commonly used regimens in published trials [17, 23].

### Systemic therapy for node-positive disease post-prostatectomy

The management of patients with high-risk, locally advanced prostate cancer found to have positive lymph nodes post-radical prostatectomy is a controversial issue. Recent data from the STAMPEDE study and NRG Oncology/RTOG 0521 study [11, 12, 24] were discussed at our update workshop held in March 2019. These studies provide data to support the use of docetaxel for six cycles or abiraterone for 2 years with radiation and ADT for 2 years in selected patients. Our panel reached consensus that ideally men found to have node-positive disease post-prostatectomy should see both a radiation oncologist and a medical oncologist for discussion regarding radiation plus systemic therapy (Table 5).

**Table 5:** High-risk features to consider adjuvant systemic therapy (docetaxel/abiraterone)

STAMPEDE criteria	At least 2 of: T 3 or 4, PSA ≥ 40 ng/ml, Gleason 8–10 Stage pT_any_ pN + M0
NRG Oncology/RTOG 0521 study criteria	Gleason 9–10 independent of PSA or T stage Gleason 7–8 and PSA ≥ 20 ng/ml with any T stage Gleason score 8 and PSA < 20 ng/ml with T stage ≥ T2

### Definitive radiation therapy for high-risk and locally advanced prostate cancer

For patients undergoing definitive radiation therapy for high-risk and locally advanced prostate cancer, techniques vary depending on local resources and expertise (Table 4). For men with access to enhanced or maximal resources in the region, several centers are now offering Intensity Modulated Radiation Therapy (IMRT) which has benefits in terms of minimizing toxicity related to treatment [25]. Recent ASTRO/ASCO/AUA guidelines recommend moderate hypofractionation across risk groups, however, the task force strongly recommended image-guided radiation therapy and avoidance of non-modulated three-dimensional conformal techniques with any hypofractionated approach [26].

For patients with high-risk disease receiving definitive radiation therapy, 36 months of ADT was not shown to be superior to 18 months of ADT. In this randomized study, overall survival was not significantly different between the two groups; however, non-inferiority could not be established [27]. Our panel currently recommend at least 18 months ADT for the majority of high-risk patients, noting that improvements in imaging and the early identification of patients with metastatic disease is likely to refine patient selection.

Investigators from The American University of Beirut Medical Center (AUBMC) have shown that nadir PSA at 0.06 ng/ml is a strong predictor of outcome in a cohort of patients with intermediate and high-risk localized prostate cancer [28].

## Management of advanced castration-sensitive/naïve prostate cancer

For patients with biochemical recurrence after radical prostatectomy and salvage radiation or primary radiation therapy, evidence-based recommendations for the timing of initiation of ADT have not been well defined. Recent clinical trials have shown a benefit in terms of prolongation of metastasis-free survival from the addition of enzalutamide, apalutamide or darolutamide in non-metastatic castration-resistant prostate cancer (nmCRPC) [29–31]. As yet, these medications are not widely available in the Middle East for this indication, however, may be considered proof of principle that earlier control of androgen receptor (AR) signaling in patients with a low burden of disease in the advanced setting may improve outcomes. Regarding the timing of initiation of ADT in men after failure of local therapy, consensus was not reached, however, 34% of panel recommend starting ADT in the majority of patients with confirmed PSA progression and 58% recommended starting ADT in a minority of selected patients for example PSA ≥ 4 ng/ml with doubling time less than 6 months. Only 8% recommend starting ADT only on detection of metastatic disease.

Our panel discussed the fact that recent trials in the setting of nmCRPC used conventional imaging for staging and that with the wider used of PET-PSMA we are seeing fewer patients with rising PSA and no radiologic evidence of metastatic disease. We discussed the limited sensitivity of PET-CT at PSA levels less than 0.5 ng/ml [22].

### Oligometastatic disease

As previously discussed, a high proportion of patients in the Middle East present with advanced prostate cancer at diagnosis. This may be due to various reasons including lack of screening, awareness and health-care access. The management of so-called “oligometastatic” prostate cancer is a controversial topic discussion at most international meetings and the subject of on-going prospective research. Our panel discussed the fact that since many patients in the region lack access to uro-oncology specialists or clinical trials, this is an area that is being addressed largely at specialist centers after multidisciplinary discussion. With the increasing use of PET-PSMA scans at diagnosis particularly in Lebanon, we are seeing more patients who may be candidates for intensification of both local and systemic therapy.

The APCCC 2017 panel did not reach consensus on the definition of oligometastatic disease with 10% of the experts stating that they did not believe that oligometastatic disease exists as a clinically meaningful entity. All of our panel in Beirut considered oligometastatic disease to be a clinically meaningful entity. There was consensus that patients with a limited number of bone and/or lymph nodes who could be treated with local therapy would constitute a clinically meaningful definition of oligometastatic prostate cancer that may influence treatment decisions (local treatment of all lesions ± systemic therapy). Where available, our panel recommends either PET-PSMA or whole-body MRI scan (depending on local expertise) for the confirmation of oligometastatic disease.

For the management of patients diagnosed with de novo oligometastatic disease with no prior treatment for prostate cancer, in the absence of clinical trials, 50% of our panelists voted for radical treatment of lesions including the primary with ADT for 24–26 months plus or minus systemic treatment with docetaxel or abiraterone, however, consensus was not reached (Table 4).

### Metastatic castration-sensitive/naïve prostate cancer (not considered oligometastatic)

For men progressing following local therapy or presenting with de novo metastatic disease, data from the CHAARTED, STAMPEDE and LATTITUDE studies have conclusively demonstrated a survival benefit from the addition of systemic therapy to standard ADT [11–14]. It is clear from the data that patients with a higher disease burden derive the greatest benefit from additional systemic therapy; however, the definition of high-volume disease has not been consistent across trials. Our panel considered that a practical definition of high-volume metastatic disease for multidisciplinary decision making is the definition used in the CHAARTED study of visceral metastasis or ≥ 4 bone lesions with ≥ 1 beyond the vertebral bodies and pelvis with the caveat that any imaging modality can be used.

We reached consensus that for men suitable for chemotherapy, docetaxel or abiraterone in addition to ADT should be recommended for castration-sensitive/naïve patients with high-volume metastatic disease as defined above (either de novo at diagnosis or at relapse following local therapy).

The cost-effectiveness of abiraterone plus prednisone until progression versus six cycles of docetaxel was discussed. No formal head-to-head comparisons have been made, however, the authors of the STAMPEDE study have published an indirect comparison between patients who were randomized to either the ADT plus docetaxel arm of the trial and the ADT plus abiraterone arm of the trial during the same time period. The investigators report no difference in overall survival between the two groups [32]. Despite the fact that generic forms of abiraterone will soon be available in the region, six cycles of docetaxel are currently significantly more cost-effective compared to abiraterone plus prednisone until progression [33]. The panel considered this to be the treatment of choice for patients who are candidates for chemotherapy.

For patients with low-volume disease as defined by the CHAARTED study, the choice of additional systemic therapy is more controversial. Long-term survival analysis of the CHAARTED study confirmed a significant survival benefit associated with docetaxel for patients with high-volume disease, however, no survival benefit was observed for patients with low-volume disease. In view of these data, many consider the evidence base to be stronger for the use of abiraterone for low-volume hormone-sensitive metastatic disease [11, 13, 34]. In view of the associated cost and limited access to this treatment in most of the region, our panel recommend that treatment with docetaxel should be considered, particularly for patients with poor adverse prognostic factors such as high Gleason grade group [32] (Table 6).

**Table 6:** Resource-stratified recommendations for the treatment of castration-sensitive/naïve advanced prostate cancer

Resource-level	Oligometastatic	Low-volume metastatic disease (not considered oligometastatic)	High-volume metastatic disease
Basic/limited	ADT—surgical/medical Consider radiation to prostate if local treatment has not been given	ADT—surgical/medical Consider radiation to prostate if local treatment has not been given	ADT—surgical/medical, consider docetaxel 6 cycles
Enhanced/maximal	Consider PET-PSMA/whole-body MRI Radiation to prostate if local treatment has not been given Consider radiation to metastatic lesions + ADT minimum 24–36 months Consider abiraterone 2 years with radiation or docetaxel 6 cycles	Radiation to prostate if local treatment has not been given ADT—lifelong (surgical/medical) Consider abiraterone until progression (if available) or docetaxel 6 cycles	No local therapy indicated unless for palliation ADT—lifelong (surgical/medical) Consider docetaxel 6 cycles (preferred in terms of cost-effectiveness) or abiraterone until progression if available

Recently published data from the STAMPEDE study have also clarified the role of radiation to the prostate in patients with advanced disease. Local radiation did not improve survival for unselected patients; however, a prespecified analysis showed that survival was improved (from 73 to 81% at 3 years) in those with a low metastatic burden [10].

Our panel reached consensus that baseline imaging and follow-up imaging at PSA nadir/completion of six cycles of docetaxel should be performed with further imaging at progression defined by confirmed PSA rise or clinical progression. Next-generation imaging was recommended if available, however, the standard of care remains CT and bone scans.

## Management of castration-resistant prostate cancer

Access to treatment modalities for metastatic castration-resistant prostate cancer (mCRPC) in the region varies according to country and financial coverage. In Lebanon, the ministry of public health covers abiraterone or enzalutamide (but not sequential therapy), docetaxel and cabazitaxel for eligible patients. We are not aware of any data suggesting excess toxicity or frequent need for dose modifications for men in the region compared to published data. Sipuleucel-T and radium-223 are not available in the region. Lutetium-PSMA therapy is available in Lebanon, however, since this is not FDA or EMEA approved it is not covered by third-party payers.

A randomized phase II study has reported non-inferiority in terms of PSA metrics between low-dose abiraterone 250 mg with a low-fat meal compared to standard dosing with 1000 mg fasting in patients CRPC [35]. Additional studies are required to assess the long-term efficacy of the strategy, however in resource-limited environments this could potentially be considered.

Our panel agreed with the APCCC 2017 consensus that asymptomatic men with mCRPC should receive abiraterone or enzalutamide as first-line treatment whether they had received ADT alone (87%) or ADT plus docetaxel in the castration-naïve setting (86%). This was qualified by the recommendation that in men with progression less than 6 months following the completion of 6 cycles of docetaxel in the castration-naïve setting, cabazitaxel could be considered. The panel also discussed the need to consider biopsy in patients progressing with visceral disease, particularly with a low PSA who may be developing androgen-independent disease and in some cases neuro-endocrine differentiation [36].

For asymptomatic men with mCRPC and progression on abiraterone or enzalutamide, the panel preferred taxane chemotherapy as a second-line option (56%), however, consensus was not reached.

We reached consensus that for symptomatic patients with acquired resistance to first-line abiraterone/enzalutamide, taxane chemotherapy should be offered (100%), and that cabazitaxel should be offered as third-line treatment for the majority of men with mCRPC progressing following second-line docetaxel and prior treatment with abiraterone/enzalutamide (81%). The panel discussed the possible use of docetaxel re-challenge in selected patients with mCRPC when cabazitaxel is not available (Table 7).

**Table 7:** Resource-stratified recommendations for the treatment of castration-resistant prostate cancer

Resource-level	Asymptomatic mCRPC	Symptomatic mCRPC	Second-line mCRPC	Third-line mCRPC
Basic/limited	Docetaxel	Docetaxel	Docetaxel re-challenge in selected patients Supportive care	Supportive care
Enhanced/maximal	Abiraterone/enzalutamide Docetaxel	Docetaxel Abiraterone/enzalutamide	Docetaxel/cabazitaxel Abiraterone/Enzalutamide Consider biopsy—if low PSA/visceral disease	Cabazitaxel Consider biopsy—if low PSA/visceral disease Consider PSMA-based theranostics if available

Our panel preferred baseline imaging and follow-up imaging at PSA nadir and again at progression, however, consensus was not reached and some opted for baseline imaging only and monitoring by PSA alone with imaging at progression. Next-generation imaging was preferred if available, otherwise CT and bone scan remain standard imaging modalities. Our panel reflected that in many cases, lines of therapy are switched based on PSA progression alone without imaging; however, ideally patient should be monitored by cross-sectional imaging.

### Use of osteoclast-targeted therapy

Our panel reflected that the monitoring of bone health for patients with prostate cancer treated with ADT in the region is sub-optimal. A useful resource is the FRAX online calculator (https://www.sheffield.ac.uk/FRAX/) that is freely available and has been validated in several middle-eastern populations [37]. All patients on ADT should be prescribed calcium and vitamin D supplementation with monitoring for osteoporosis.

The RANK-ligand inhibitor denosumab and bisphosphonate zoledronic acid are both available in the region; however, denosumab is only approved for the treatment of osteoporosis and not advanced cancer with bone metastasis in some countries. Neither drug has been shown to improve survival or influence progression-free survival, however, in the setting of mCRPC both drugs can protect against skeletal-related events (SREs) [38–40]. There is no evidence to support the use of osteoclast-targeted therapy in the non-metastatic setting or metastatic castration-naïve patients for SRE prevention (Table 8).

**Table 8:** Resource-stratified recommendations for monitoring bone health and the use of osteoclast-targeted therapy

Resource-level	Monitoring bone heath on ADT	Non-osteoporotic patients with localized/advanced HSPC	Non-osteoporotic patients with mCRPC (normal renal function)
Basic/limited	DXA scan after 2 years on ADT	No osteoclast-directed therapy	Consider zoledronic acid 4 mg IV every 3 months
Enhanced/maximal	DXA scan at start of ADT and every 2 years on treatment	No osteoclast-directed therapy	Consider zoledronic acid 4 mg IV every 4–12 weeks Consider denosumab 120 mg s/c every 4 weeks

Our panel reached consensus that osteoclast-targeted therapy for SRE prevention should be recommend in the majority of patient with mCRPC and bone metastasis. A dental check-up should be performed prior to starting therapy to decrease the risk of osteonecrosis of the jaw. Most of the panel recommend zoledronic acid for 2 years which can be given every 3 months and may be more cost-effective that denosumab that should be given every 4 weeks continuously [41].

## Conclusion

This satellite meeting of the APCCC–MEPCC is the first regional consensus on prostate cancer management in the Middle East that has attempted to set out recommendations based on availability of local resources and expertise. Our group acknowledges that participants were largely from Lebanon and from tertiary referral centers in the region. This reflects our management approach, in particular our use of novel diagnostic imaging modalities such as PET-PSMA which is widely available in Lebanon. We hope this review of controversial issues in prostate cancer management will be useful to the non-specialist urologist or oncologist practicing in all areas with limited access to specialist multidisciplinary teams, diagnostic and treatment modalities.

## Electronic supplementary material

Below is the link to the electronic supplementary material.

Supplementary material 1 (DOCX 62 kb)
