# Supplementary material for: Management of patients with high-risk and advanced prostate cancer in the Middle East: resource-stratified consensus recommendations
Source: World J Urol. 2019 Jul 11;38(3):681–93. doi: 10.1007/s00345-019-02872-x (PMC7064460; doi:10.1007/s00345-019-02872-x)
Supplement: Supplementary file 1 — Supplementary material 1 (DOCX 62 kb) [file 345_2019_2872_MOESM1_ESM.docx]

Advanced Prostate Cancer Consensus Conference Beirut Satellite Questions

November 3-4 2017

**Session 1**

**High risk locally advanced**

**Management of high-risk and locally-advanced M0 prostate cancer**

|  | 1.1 Do you recommend lymph node dissection in men with cN0 cM0 high-risk prostate cancer undergoing prostatectomy? | | | | |
| --- | --- | --- | --- | --- | --- |
|  | | BEIRUT MEETING (%) | Percentage of eligible votes Beirut | ST GALLEN MEETING (%) | Votes in ST G (N 50) |
| Yes, in the majority of patients | | 76 % | 76 | 84% | 38 |
| In a minority of selected patients | | 19% | 19 | 9% | 4 |
| No | | 2% | 2 | 5% | 2 |
| Abstain | | 2% | 2 | 2% | 1 |
| Unqualified to answer | | 0% |  |  | 5 |

|  | 1.2 What is the minimum number of lymph nodes removed you consider adequate in the majority of men with cN0 cM0 high-risk prostate cancer? | | | | |
| --- | --- | --- | --- | --- | --- |
|  | | BEIRUT MEETING (%) | Percentage of eligible votes Beirut | ST GALLEN MEETING (%) | Votes in ST G (N 49) |
| <5 | | 2 | 2 | 0 | 0 |
| 5-10 | | 26 | 28 | 15 | 7 |
| 11-19 | | 45 | 49 | 49 | 22 |
| ≥20 | | 19 | 21 | 27 | 12 |
| Abstain | | 0 | 0 | 9 | 4 |
| Unqualified to answer | | 7 |  |  | 4 |

|  | 1.3 What imaging test is sufficient to “exclude” distant metastases in high-risk and locally-advanced prostate cancer? | | | | |
| --- | --- | --- | --- | --- | --- |
|  | | BEIRUT MEETING (%) | Percentage of eligible votes Beirut | ST GALLEN MEETING (%) | Votes in ST G (N 49) |
| Bone scintigraphy alone | | 2 | 2 | 0 | 0 |
| CT alone | | 0 |  | 0 | 0 |
| Combination of bone scintigraphy and CT | | 17 | 18 | 41 | 20 |
| Whole body MRI | | 14 | 14 | 10 | 5 |
| PET-CT (PSMA, Choline or FACBC (Fluciclovine)) | | 64 | 66 | 37 | 18 |
| Abstain | | 0 |  | 12 | 6 |
| Unqualified to answer | | 2 |  | 0 | 0 |

**Adjuvant radiation therapy for localized prostate cancer**

|  | 1.4 In men post-prostatectomy without lymph node involvement on surgical pathology (pN0), with undetectable postoperative PSA and who have recovered urinary continence, do you recommend adjuvant radiation therapy in in case of: Seminal vesicle involvement? | | | | |
| --- | --- | --- | --- | --- | --- |
|  | | BEIRUT MEETING (%) | Percentage of eligible votes Beirut | ST GALLEN MEETING (%) | Votes in ST G (N 51) |
| Yes, in the majority of patients | | 56 | 58 | 38 | 19 |
| Only if margin positive | | 34 | 35 | 32 | 16 |
| No | | 7 | 7 | 26 | 13 |
| Abstain | | 0 |  | 4 | 2 |
| Unqualified to answer | | 2 |  |  | 1 |

|  | 1.5 In men post-prostatectomy without lymph node involvement on surgical pathology (pN0), with undetectable postoperative PSA and who have recovered urinary continence, do you recommend adjuvant radiation therapy in in case of: Positive surgical margins | | | | |
| --- | --- | --- | --- | --- | --- |
|  | | BEIRUT MEETING (%) | Percentage of eligible votes Beirut | ST GALLEN MEETING (%) | Votes in ST G (N 52) |
| Yes, in the majority of patients | | 81 | 81 | 45 | 25 |
| Only if multifocal or extensive margins | | 12 | 12 | 27 | 14 |
| No | | 7 | 7 | 21 | 11 |
| Abstain | | 0 |  | 4 | 2 |
| Unqualified to answer | | 0 |  | 0 | 0 |

|  | 1.6 In men post-prostatectomy without lymph node involvement on surgical pathology (pN0), with undetectable postoperative PSA and who have recovered urinary continence, do you recommend adjuvant radiation therapy in in case of: Gleason 8-10 or Gleason Grade Group 4 or 5? | | | | |
| --- | --- | --- | --- | --- | --- |
|  | | BEIRUT MEETING (%) | Percentage of eligible votes Beirut | ST GALLEN MEETING (%) | Votes in ST G (N 51) |
| Yes, in the majority of patients | | 36 | 36 | 20 | 10 |
| In a minority of selected patients | | 15 | 15 | 23 | 12 |
| No | | 46 | 46 | 55 | 28 |
| Abstain | | 3 | 3 | 2 | 1 |
| Unqualified to answer | | 0 |  | 0 | 0 |

|  | 1.7 If you recommend adjuvant radiation therapy in men with high-risk pN0 disease post-prostatectomy, what field of radiation therapy do you recommend in the majority of men? | | | | |
| --- | --- | --- | --- | --- | --- |
|  | | BEIRUT MEETING (%) | Percentage of eligible votes Beirut | ST GALLEN MEETING (%) | Votes in ST G (N 51) |
| Prostatic bed only | | 38 | 42 | 41 | 15 |
| Prostatic bed plus whole pelvis | | 38 | 42 | 51 | 19 |
| I do not recommend adjuvant radiation therapy | | 14 | 16 | 8 | 9 |
| Abstain | | 7 |  |  | 3 |
| Unqualified to answer | | 2 |  |  | 5 |

|  | 1.8 If you recommend adjuvant radiation therapy in men with high-risk pN0 disease post-prostatectomy, do you recommend adding ADT? | | | | |
| --- | --- | --- | --- | --- | --- |
|  | | BEIRUT MEETING (%) | Percentage of eligible votes Beirut | ST GALLEN MEETING (%) | Votes in ST G (N 51) |
| Yes, in the majority of patients | | 59 | 70 | 36 | 15 |
| In a minority of selected patients | | 10 | 12 | 32 | 13 |
| No | | 15 | 18 | 32 | 13 |
| Abstain (I do not recommend adjuvant radiation therapy) | | 17 |  |  | 9 |
| Unqualified to answer | | 0 |  |  | 1 |

|  | 1.9 In which subgroup of men with high-risk pN0 prostate cancer do you recommend ADT with adjuvant radiation therapy? | | | | |
| --- | --- | --- | --- | --- | --- |
|  | | BEIRUT MEETING (%) | Percentage of eligible votes Beirut | ST GALLEN MEETING (%) | Votes in ST G (N 51) |
| pT stage ≥3b | | 13 | 16 | 28 | 8 |
| Gleason 8-10 or Gleason Grade Group 4 or 5 | | 5 | 6 | 3 | 1 |
| In both subgroups (pT stage ≥3b and/or Gleason score ≥8) | | 63 | 78 | 69 | 20 |
| I do not recommend concurrent ADT | | 13 |  |  | 14 |
| Abstain | | 3 |  |  | 6 |
| Unqualified to answer | | 3 |  |  | 2 |

|  | 1.10 If you recommend adding ADT to adjuvant radiation therapy in men with pN0 post-prostatectomy, what duration of ADT do you recommend in the majority of men? | | | | |
| --- | --- | --- | --- | --- | --- |
|  | | BEIRUT MEETING (%) | Percentage of eligible votes Beirut | ST GALLEN MEETING (%) | Votes in ST G (N 51) |
| 3-6 months | | 29 | 32 | 39 | 13 |
| 6-12 months | | 13 | 15 | 43 | 14 |
| 18-36 months | | 47 | 53 | 18 | 6 |
| Lifelong | | 0 | 0 | 0 | 0 |
| Abstain (I do not recommend adjuvant radiation therapy and/or addition of ADT) | | 8 |  |  | 17 |
| Unqualified to answer | | 3 |  |  | 1 |

|  | 1.11 Do you recommend adjuvant radiation therapy in men with pN1 disease (adequate sampling) and no local adverse factors (no pT3b, no R1) and undetectable postoperative PSA and who have recovered urinary continence? | | | | |
| --- | --- | --- | --- | --- | --- |
|  | | BEIRUT MEETING (%) | Percentage of eligible votes Beirut | ST GALLEN MEETING (%) | Votes in ST G (N 52) |
| Yes, in the majority of patients | | 62 | 62 | 26 | 13 |
| In a minority of selected patients | | 19 | 19 | 29 | 15 |
| No | | 19 | 19 | 43 | 22 |
| Abstain | | 0 |  | 2 | 1 |
| Unqualified to answer | | 0 |  |  | 1 |

|  | 1.12 If you recommend adjuvant radiation therapy in men with pN1 disease (adequate sampling) post-prostatectomy, what field of radiation therapy do you recommend in the majority of men? | | | | |
| --- | --- | --- | --- | --- | --- |
|  | | BEIRUT MEETING (%) | Percentage of eligible votes Beirut | ST GALLEN MEETING (%) | Votes in ST G (N 52) |
| Prostatic bed only | | 2 | 2 | 0 | 0 |
| Prostatic bed plus whole pelvis | | 80 | 92 | 97 | 32 |
| Other field definition | | 5 | 6 | 3 | 1 |
| Abstain (including I do not recommend adjuvant radiation therapy) | | 7 |  |  | 17 |
| Unqualified to answer | | 5 |  |  | 2 |

|  | 1.13 In which subgroup of men with pN1 (adequate sampling) post-prostatectomy do you recommend adjuvant radiation therapy? | | | | |
| --- | --- | --- | --- | --- | --- |
|  | | BEIRUT MEETING (%) | Percentage of eligible votes Beirut | ST GALLEN MEETING (%) | Votes in ST G (N 52) |
| In all patients | | 59 | 60 | 17 | 6 |
| In men with 1 or 2 positive lymph nodes in the presence of  intermediate- or high-grade, non-organ-confined disease and in those with 3 to 4 lymph nodes | | 27 | 29 | 50 | 17 |
| In patients with ≤2 positive lymph nodes independent of  grade and T-stage | | 3 | 3 | 15 | 5 |
| In patients with ≤3 positive lymph nodes independent of  grade and T-stage | | 3 | 3 |  | 1 |
| In patients with ≤4 positive lymph nodes independent of  grade and T-stage | | 5 | 5 | 15 | 5 |
| Abstain (including I do not recommend adjuvant  radiation therapy) | | 3 |  |  | 18 |
| Unqualified to answer | | 0 |  |  | 0 |

|  | 1.14 If you recommend adjuvant radiation therapy and ADT in the majority of men with pN1 disease what duration of ADT do you recommend? | | | | |
| --- | --- | --- | --- | --- | --- |
|  | | BEIRUT MEETING (%) | Percentage of eligible votes Beirut | ST GALLEN MEETING (%) | Votes in ST G (N 50) |
| 3-6 months | | 5 | 5 | 11 | 4 |
| 6-12 months | | 16 | 17 | 30 | 11 |
| 18-36 months | | 70 | 73 | 57 | 21 |
| Lifelong | | 5 | 5 | 2 | 1 |
| Abstain (I do not recommend adjuvant radiation therapy and/or addition of ADT) | | 3 |  |  | 13 |
| Unqualified to answer | | 0 |  |  | 0 |

**Salvage Radiation therapy for isolated rising PSA alone after prostatectomy**

|  | 1.15 At what confirmed PSA level do you do you recommend starting salvage radiation therapy in the majority of men with isolated rising PSA alone after prostatectomy? | | | | |
| --- | --- | --- | --- | --- | --- |
|  | | BEIRUT MEETING (%) | Percentage of eligible votes Beirut | ST GALLEN MEETING (%) | Votes in ST G (N 51) |
| <0.1 ng/mL | | 0 | 0 | 4 | 2 |
| 0.1 ng/mL | | 8 | 8 | 38 | 19 |
| 0.2 ng/mL | | 53 | 54 | 44 | 22 |
| 0.5 ng/mL | | 32 | 33 | 10 | 5 |
| 1.0 ng/mL | | 5 | 5 | 0 | 0 |
| >1 ng/mL | | 0 | 0 | 0 | 0 |
| Abstain | | 0 |  | 4 | 2 |
| Unqualified to answer | | 3 |  |  | 1 |

| 1.16 Do you recommend adding ADT in combination with salvage radiation therapy? | | | |
| --- | --- | --- | --- |
|  | BEIRUT MEETING (%) | ST GALLEN MEETING (%) | Votes in ST G (N 50) |
| Yes, in the majority of patients | 83 | 61 | 30 |
| In a minority of selected patients e.g. based on PSA level and PSA-DT | 8 | 29 | 14 |
| No | 8 | 10 | 5 |
| Abstain | 0 |  | 0 |
| Unqualified to answer | 0 |  | 1 |

|  | 1.17 If you recommend adding ADT in combination with salvage radiation therapy which duration of ADT do you recommend in the majority of men? | | | | |
| --- | --- | --- | --- | --- | --- |
|  | | BEIRUT MEETING (%) | Percentage of eligible votes Beirut | ST GALLEN MEETING (%) | Votes in ST G (N 51) |
| 3-6 months | | 17 | 18 | 34 | 15 |
| 6-12 months | | 11 | 11 | 41 | 18 |
| 18-36 months | | 56 | 57 | 25 | 11 |
| Lifelong | | 14 | 14 | 0 | 0 |
| Abstain (Including I do not recommend adding ADT) | | 3 |  |  | 6 |
| Unqualified to answer | | 0 |  |  | 1 |

**Session 2**

**Oligometastatic + Bone Health**

**Definition of oligometastatic disease**

|  | | 2.1 A clinically meaningful definition of oligometastatic prostate cancer that influences treatment decision (local treatment of all lesions +/- systemic therapy) includes: | | | |
| --- | --- | --- | --- | --- | --- |
|  | BEIRUT MEETING (%) | | Percentage of eligible votes Beirut | ST GALLEN MEETING (%) | Votes in ST G (N 51) |
| Only patients with a limited number of bone and/or lymph  nodes metastases that can be treated with local therapy | 81 | | 81 | 61 | 31 |
| Only patients with a limited number of lymph node metastases  that can be treated with local therapy | 13 | | 13 | 10 | 5 |
| A limited number of any metastases (including visceral) | 6 | | 6 | 13 | 7 |
| I do not believe oligometastatic prostate cancer exists as a  clinically meaningful entity | 0 | | 0 | 10 | 5 |
| Abstain | 0 | | 0 | 6 | 3 |
| Unqualified to answer | 0 | |  | 0 | 0 |

**De novo oligometastatic disease (no prior prostate treatment)**

|  | | 2.2 Which treatment do you recommend in men with newly-diagnosed oligometastatic prostate cancer with an untreated primary? | | | |
| --- | --- | --- | --- | --- | --- |
|  | BEIRUT MEETING (%) | | Percentage of eligible votes Beirut | ST GALLEN MEETING (%) | Votes in ST G (N 51) |
| Lifelong ADT +/- Docetaxel | 13 | | 13 | 25 | 13 |
| Radical local treatment of all lesions including the primary  (surgery or RT) without ADT or Docetaxel | 0 | | 0 | 8 | 4 |
| Radical local treatment of all lesions including the primary  (surgery or RT) + ADT 6-12m +/- Docetaxel | 17 | | 17 | 22 | 11 |
| Radical local treatment of all lesions including the primary  (surgery or RT) + ADT 24-36m +/- Docetaxel | 50 | | 59 | 31 | 16 |
| Radical local treatment of all lesions including the primary  (surgery or RT) + lifelong ADT +/- Docetaxel | 20 | | 29 | 8 | 4 |
| Abstain | 0 | |  | 6 | 3 |
| Unqualified to answer | 0 | |  | 0 | 0 |

|  | | 2.3 In men with newly-diagnosed oligometastatic prostate cancer and an untreated primary what do you recommend for treatment of the primary? | | | |
| --- | --- | --- | --- | --- | --- |
|  | BEIRUT MEETING (%) | | Percentage of eligible votes Beirut | ST GALLEN MEETING (%) | Votes in ST G (N 52) |
| Radical prostatectomy (RP) | 13 | | 14 | 22 | 9 |
| Radiation therapy (RT) | 19 | | 20 | 45 | 19 |
| Either RT or RP | 63 | | 66 | 31 | 13 |
| Other | 0 | | 0 | 2 | 1 |
| Abstain (including I do not recommend radical local  treatment of the primary) | 6 | |  |  | 10 |
| Unqualified to answer | 0 | |  |  | 0 |

|  | | 2.4 What treatment do you recommend in the majority of asymptomatic men developing oligometastatic recurrent castration-sensitive/naive prostate cancer after local treatment of the primary with curative intent (+/- salvage radiation therapy)? | | | |
| --- | --- | --- | --- | --- | --- |
|  | BEIRUT MEETING (%) | | Percentage of eligible votes Beirut | ST GALLEN MEETING (%) | Votes in ST G (N 50) |
| Lifelong ADT +/- Docetaxel | 22 | | 22 | 32 | 16 |
| Radical local treatment of all lesions (surgery or RT)  without ADT or Docetaxel | 4 | | 4 | 12 | 6 |
| Radical local treatment of all lesions (surgery or RT)  + ADT 6-12m +/- Docetaxel | 7 | | 7 | 30 | 15 |
| Radical local treatment of all lesions (surgery or RT)  + ADT 24-36m +/- Docetaxel | 48 | | 48 | 18 | 9 |
| Radical local treatment of lesions (surgery or RT) + lifelong ADT +/- Docetaxel | 19 | | 19 | 4 | 2 |
| Abstain | 0 | | 0 | 4 | 2 |
| Unqualified to answer | 0 | |  | 0 | 0 |

**Oligometastatic recurrence in castration-sensitive/naive prostate cancer patients with a rising PSA after local treatment (EBRT or RP ± EBRT) with curative intent (+/- salvage radiation therapy)**

|  | | 2.5 What treatment do you recommend if you consider metastasis directed therapy in men with oligometastatic recurrent castration-sensitive/naive prostate cancer that is limited to lymph node metastases in the pelvis after local treatment with curative intent (+/- salvage radiation therapy)? | | | |
| --- | --- | --- | --- | --- | --- |
|  | BEIRUT MEETING (%) | | Percentage of eligible votes Beirut | ST GALLEN MEETING (%) | Votes in ST G (N 52) |
| Salvage lymph node dissection | 7 | | 7 | 23 | 10 |
| Salvage lymph node dissection + whole pelvis radiotherapy | 40 | | 41 | 19 | 8 |
| Focal radiotherapy to the suspicious nodes | 0 | | 0 | 16 | 7 |
| Whole pelvis radiotherapy +/- boost to the suspicious nodes | 50 | | 52 | 42 | 18 |
| Abstain (including I do not believe that the oligometastatic  recurrent state is a clinically meaningful entity) | 3 | |  |  | 8 |
| Unqualified to answer | 0 | |  |  | 1 |

**Rising PSA on ADT (mCRPC) and oligometastatic disease**

|  | | 2.6 What treatment do you recommend in men with oligometastatic CRPC? | | | |
| --- | --- | --- | --- | --- | --- |
|  | BEIRUT MEETING (%) | | Percentage of eligible votes Beirut | ST GALLEN MEETING (%) | Votes in ST G (N 52) |
| Standard of care (continue ADT and add additional  systemic therapy) | 35 | | 37 | 44 | 21 |
| Local treatment of all lesions and discontinue ADT | 0 | | 0 | 2 | 1 |
| Local treatment of all lesions and continue ADT | 6 | | 6 | 25 | 12 |
| Local treatment of all lesions and continue ADT and add  additional systemic treatment for mCRPC | 55 | | 57 | 29 | 14 |
| Abstain (including I do not believe that oligometastatic  prostate cancer is a clinically meaningful entity in mCRPC) | 3 | |  |  | 4 |
| Unqualified to answer | 0 | |  |  | 0 |

|  | | 2.7 In men with potentially de novo oligometastatic disease what imaging do you recommend to confirm this diagnosis (apart from local staging)? | | | |
| --- | --- | --- | --- | --- | --- |
|  | BEIRUT MEETING (%) | | Percentage of eligible votes Beirut | ST GALLEN MEETING (%) | Votes in ST G (N 50) |
| CT and/or MRI and bone scintigraphy | 14 | | 14 | 26 | 13 |
| PET-CT (PSMA, Choline or FACBC (Fluciclovine)) | 71 | | 71 | 34 | 17 |
| WB-MRI | 0 | | 0 | 4 | 2 |
| Either PET-CT or WB-MRI (next generation imaging) | 14 | | 14 | 34 | 17 |
| Abstain | 0 | | 0 | 2 | 1 |
| Unqualified to answer | 0 | |  | 0 | 0 |

**Best use of osteoclast-targeted therapy for reducing risk of SRE/SSE (not for osteoporosis)**

**Osteoclast-targeted therapy in men with M1 castration-naïve prostate cancer**

|  | | 2.8 Do you **recommend** zoledronic acid (4mg every 3-4 weeks) in castration-sensitive M1 patients with bone metastases? | | | |
| --- | --- | --- | --- | --- | --- |
|  | BEIRUT MEETING (%) | |  | ST GALLEN MEETING (%) |  |
| Yes, in the majority of patients | 53 | | 57 | 2.8 |  |
| In a minority of selected patients | 13 | | 14 | 16.7 |  |
| No | 27 | | 29 | 80.6 |  |
| Abstain | 3 | |  |  |  |
| Unqualified to answer | 3 | |  |  |  |

|  | | 2.9 Do you **recommend** denosumab (120mg every 4 weeks) in castration-sensitive M1 patients with bone metastases? | | | |
| --- | --- | --- | --- | --- | --- |
|  | BEIRUT MEETING (%) | | Percentage of eligible votes Beirut | ST GALLEN MEETING (%) |  |
| Yes, in the majority of patients | 30 | | 32 | 2.9 |  |
| In a minority of selected patients | 22 | | 24 | 17.6 |  |
| No | 41 | | 44 | 79.4 |  |
| Abstain | 4 | |  |  |  |
| Unqualified to answer | 4 | |  |  |  |

**Best use of osteoclast-targeted therapy for SRE/SSE prevention for mCRPC (NOT for osteoporosis/bone loss)**

|  | | 2.10 Do you **recommend** an osteoclast-targeted therapy for SRE prevention in CRPC patients **with** bone metastases? | | | |
| --- | --- | --- | --- | --- | --- |
|  | BEIRUT MEETING (%) | | Percentage of eligible votes Beirut | ST GALLEN MEETING (%) |  |
| Yes, in the majority of patients | 65 | | 68 | 61.8 |  |
| In a minority of selected patients | 15 | | 16 | 35.4 |  |
| No | 15 | | 16 | 5.9 |  |
| Abstain | 0 | | 0 |  |  |
| Unqualified to answer | 4 | |  |  |  |

|  | | 2.11 Do you **recommend** a dental check for CRPC patients with bone metastases prior to starting an osteoclast-targeted therapy? | | | |
| --- | --- | --- | --- | --- | --- |
|  | BEIRUT MEETING (%) | | Percentage of eligible votes Beirut | ST GALLEN MEETING (%) |  |
| Yes, in the majority of patients | 79 | | 82 | 75.8 |  |
| In a minority of selected patients | 10 | | 11 | 213.2 |  |
| No | 7 | | 7 | 3 |  |
| Abstain | 0 | | 0 | 3 |  |
| Unqualified to answer | 3 | |  |  |  |

|  | | 2.12 When you use osteoclast-targeted therapy (zoledronic acid or denosumab) in men with mCRPC and bone metastases, what treatment duration do you recommend? | | | |
| --- | --- | --- | --- | --- | --- |
|  | BEIRUT MEETING (%) | | Percentage of eligible votes Beirut | ST GALLEN MEETING (%) | Votes in ST G (N 50) |
| Approximately 2 years | 34 | | 38 | 68 | 30 |
| Indefinitely | 55 | | 62 | 32 | 14 |
| Abstain (including I do not use  osteoclast-targeted therapy in this setting) | 3 | |  |  | 6 |
| Unqualified to answer | 7 | |  |  | 0 |

**Session 3**

**Castrate-naïve prostate cancer**

**When to start ADT? (Post-prostatectomy (+/- RT) or Post Radical RT)**

|  | | 3.1 In men with non-metastatic disease and confirmed rising PSA (post-local therapy +/- salvage local RT), do you recommend starting ADT? | | | |
| --- | --- | --- | --- | --- | --- |
|  | BEIRUT MEETING (%) | | Percentage of eligible votes Beirut | ST GALLEN MEETING (%) | Votes in ST G (N 51) |
| Yes, in the majority of patients | 34 | | 34 | 21 | 11 |
| In a minority of selected patients e.g. PSA ≥4ng/ml and rising with  doubling time less than 6 months OR PSA ≥20ng/ml (STAMPEDE inclusion criteria) | 58 | | 58 | 65 | 33 |
| No, I only recommend ADT after detection of metastatic disease | 8 | | 8 | 12 | 6 |
| Abstain | 0 | | 0 | 2 | 1 |
| Unqualified to answer | 0 | |  | 0 | 0 |

**Castration-sensitive/naive metastatic prostate cancer**

|  | | 3.2 For the purpose of treatment selection, what is the most meaningful definition of high-volume disease in castration-sensitive/naive metastatic prostate cancer? | | | |
| --- | --- | --- | --- | --- | --- |
|  | BEIRUT MEETING (%) | | Percentage of eligible votes Beirut | ST GALLEN MEETING (%) | Votes in ST G (N 51) |
| “High-volume” as defined by CHAARTED by bone scintigraphy  and CT scan (visceral metastases and/or ≥4 bone lesions with  ≥1 beyond vertebral bodies and pelvis) | 49 | | 49 | 59 | 30 |
| “High-volume” as defined by CHAARTED using  any imaging modality (including next generation imaging) | 11 | | 11 | 15 | 8 |
| Visceral (lung or liver) and/or any appendicular skeletal  involvement (SWOG) | 14 | | 14 | 6 | 3 |
| A simplified version of high volume of visceral and/or ≥4 bone lesions  regardless of distribution and imaging used | 14 | | 14 | 6 | 3 |
| I don’t think “high-volume” disease is a clinically meaningful entity | 3 | | 3 | 14 | 7 |
| Abstain | 11 | | 11 | 0 | 0 |
| Unqualified to answer | 0 | |  | 0 | 0 |

|  | | 3.3 For men who are suitable for chemotherapy: Do you recommend Docetaxel in addition to ADT in men with de novo metastatic castration-sensitive/naive prostate cancer and high volume disease as defined by CHAARTED (visceral metastases and/or ≥4 bone lesions with ≥1 beyond vertebral bodies and pelvis)? | | | |
| --- | --- | --- | --- | --- | --- |
|  | BEIRUT MEETING (%) | | Percentage of eligible votes Beirut | ST GALLEN MEETING (%) | Votes in ST G (N 50) |
| Yes, in the majority of patients | 95 | | 95 | 96 | 48 |
| In a minority of selected patients | 0 | | 0 | 4 | 2 |
| No | 5 | | 5 | 0 | 0 |
| Abstain | 0 | | 0 | 0 | 0 |
| Unqualified to answer | 0 | |  | 0 | 0 |

|  | | 3.4 Do you recommend Docetaxel in addition to ADT in men with de novo metastatic castration-sensitive/naive and low-volume disease as per CHAARTED (no visceral metastases and <4 bone lesions and only confined to axial skeleton)? | | | |
| --- | --- | --- | --- | --- | --- |
|  | BEIRUT MEETING (%) | | Percentage of eligible votes Beirut | ST GALLEN MEETING (%) | Votes in ST G (N 50) |
| Yes, in the majority of patients | 11 | | 11 | 29 | 15 |
| In a minority of selected patients | 67 | | 67 | 65 | 34 |
| No | 22 | | 22 | 6 | 3 |
| Abstain | 0 | | 0 | 0 | 0 |
| Unqualified to answer | 0 | |  | 0 | 0 |

|  | | 3.5 Do you recommend Docetaxel in addition to ADT in with metastatic castration-sensitive/naive disease relapsing after prior treatment for localized prostate cancer and with high volume disease as per CHAARTED (visceral metastases and/or ≥4 bone lesions with ≥1 beyond vertebral bodies and pelvis)? | | | |
| --- | --- | --- | --- | --- | --- |
|  | BEIRUT MEETING (%) | | Percentage of eligible votes Beirut | ST GALLEN MEETING (%) | Votes in ST G (N 50) |
| Yes, in the majority of patients | 89 | | 89 | 74 | 37 |
| In a minority of selected patients | 8 | | 8 | 24 | 12 |
| No | 3 | | 3 | 2 | 1 |
| Abstain | 0 | | 0 | 0 | 0 |
| Unqualified to answer | 0 | |  | 0 | 0 |

|  | | 3.6 Do you recommend Docetaxel in addition to ADT in with metastatic castration-sensitive/naive disease relapsing after prior treatment for localized prostate cancer with low volume bone metastases as per CHAARTED criteria (no visceral metastases and <4 bone lesions)? | | | |
| --- | --- | --- | --- | --- | --- |
|  | BEIRUT MEETING (%) | | Percentage of eligible votes Beirut | ST GALLEN MEETING (%) | Votes in ST G (N 52) |
| Yes, in the majority of patients | 19 | | 19 | 19 | 10 |
| In a minority of selected patients | 68 | | 68 | 54 | 28 |
| No | 14 | | 14 | 25 | 13 |
| Abstain | 0 | | 0 | 2 | 1 |
| Unqualified to answer | 0 | |  | 0 | 0 |

|  | | 3.7 Do you recommend Docetaxel in addition to ADT in men with castration-sensitive/naive N1 M0 prostate cancer? | | | |
| --- | --- | --- | --- | --- | --- |
|  | BEIRUT MEETING (%) | | Percentage of eligible votes Beirut | ST GALLEN MEETING (%) | Votes in ST G (N 51) |
| Yes, in the majority of patients | 19 | | 20 | 25 | 2 |
| In a minority of selected patients | 39 | | 40 | 71 | 13 |
| No | 39 | | 40 | 4 | 36 |
| Abstain | 0 | |  | 0 | 0 |
| Unqualified to answer | 3 | |  | 0 | 0 |

**Treating the prostate in men with M1 castration-sensitive/naive prostate cancer**

|  | | 3.8 In men with de novo metastatic castration-sensitive/naive high-volume prostate cancer, who are not symptomatic from the primary, do you recommend treatment of the primary tumour in addition to systemic therapy? | | | |
| --- | --- | --- | --- | --- | --- |
|  | BEIRUT MEETING (%) | | Percentage of eligible votes Beirut | ST GALLEN MEETING (%) | Votes in ST G (N 50) |
| Yes, in the majority of patients | 37 | | 37 | 10 | 5 |
| In a minority of selected patients | 31 | | 31 | 38 | 19 |
| No | 31 | | 31 | 52 | 26 |
| Abstain | 0 | | 0 | 0 | 0 |
| Unqualified to answer | 0 | |  | 0 | 0 |

|  | | 3.9 If you recommend treatment of the primary in this situation, what is your preferred treatment option in the majority of men? | | | |
| --- | --- | --- | --- | --- | --- |
|  | BEIRUT MEETING (%) | | Percentage of eligible votes Beirut | ST GALLEN MEETING (%) | Votes in ST G (N 52) |
| Radiation therapy | 56 | | 77 | 71 | 22 |
| Prostatectomy (if clinically operable cancer) | 14 | | 19 | 26 | 8 |
| Other local treatment of the primary | 3 | | 4 | 3 | 1 |
| Abstain (including “I do not recommend treatment of the primary in this situation”) | 28 | |  |  | 21 |
| Unqualified to answer | 0 | |  |  | 0 |

**Session 4**

**Castration-Resistant Prostate Cancer**

**Sequencing and combinations in mCRPC**

|  | | 4.1 What is your preferred first-line mCRPC treatment option in the majority of asymptomatic or minimally symptomatic men who did NOT receive Docetaxel in the castration-sensitive/naive setting? | | | |
| --- | --- | --- | --- | --- | --- |
|  | BEIRUT MEETING (%) | | Percentage of eligible votes Beirut | ST GALLEN MEETING (%) | Votes in ST G (N 51) |
| Abiraterone or Enzalutamide | 87 | | 87 | 86 | 44 |
| Cabazitaxel | 0 | | 0 | 0 | 0 |
| Docetaxel | 13 | | 13 | 6 | 3 |
| Platinum based chemotherapy | 0 | | 0 | 0 | 0 |
| No preferred option | 0 | | 0 | 0 | 0 |
| Abstain | 0 | | 0 | 0 | 0 |
| Unqualified to answer | 0 | |  | 0 | 0 |

|  | | 4.2 What is your preferred first-line mCRPC treatment option in the majority of symptomatic men who did NOT receive Docetaxel in the castration-sensitive/naive setting? | | | |
| --- | --- | --- | --- | --- | --- |
|  | BEIRUT MEETING (%) | | Percentage of eligible votes Beirut | ST GALLEN MEETING (%) | Votes in ST G (N 50) |
| Abiraterone or Enzalutamide | 43 | | 42 | 52 | 26 |
| Cabazitaxel | 0 | | 0 | 0 | 0 |
| Docetaxel | 53 | | 53 | 46 | 23 |
| Platinum based chemotherapy | 0 | | 0 | 0 | 0 |
| No preferred option | 3 | | 3 | 1 | 1 |
| Abstain | 0 | | 0 | 0 | 0 |
| Unqualified to answer | 0 | |  | 0 | 0 ( 1 answered Radium 23) |

|  | | 4.3 What is your preferred first-line mCRPC treatment option in the majority of asymptomatic or minimally symptomatic men who did receive Docetaxel in the castration-sensitive/naive setting? | | | |
| --- | --- | --- | --- | --- | --- |
|  | BEIRUT MEETING (%) | | Percentage of eligible votes Beirut | ST GALLEN MEETING (%) | Votes in ST G (N 49) |
| Abiraterone or Enzalutamide | 86 | | 86 | 90 | 44 |
| Cabazitaxel | 14 | | 14 | 2 | 1 |
| Docetaxel | 0 | | 0 | 2 | 1 |
| Platinum based chemotherapy | 0 | | 0 | 0 | 0 |
| No preferred option | 0 | | 0 | 0 | 0 |
| Abstain | 0 | | 0 | 0 | 0 |
| Unqualified to answer | 0 | |  | 0 | 0 ( 3 sipuleucel T) |

|  | | 4.4 What is your preferred first-line mCRPC treatment option in the majority of symptomatic men who did receive Docetaxel in the castration-sensitive/naive setting? | | | |
| --- | --- | --- | --- | --- | --- |
|  | BEIRUT MEETING (%) | | Percentage of eligible votes Beirut | ST GALLEN MEETING (%) | Votes in ST G (N 48 |
| Abiraterone or Enzalutamide | 55 | | 55 | 73 | 35 |
| Cabazitaxel | 38 | | 38 | 19 | 9 |
| Docetaxel | 3 | | 3 | 6 | 3 |
| Platinum based chemotherapy | 3 | | 3 | 0 | 0 |
| No preferred option | 0 | | 0 | 0 | 0 |
| Abstain | 0 | | 0 | 0 | 0 |
| Unqualified to answer | 0 | |  | 0 | 0 (1 Radium-233 ) |

|  | | 4.5 If you have to choose between Abiraterone and Enzalutamide what is your preferred first-line choice for men with mCRPC with no contraindication to either drug? | | | |
| --- | --- | --- | --- | --- | --- |
|  | BEIRUT MEETING (%) | | Percentage of eligible votes Beirut | ST GALLEN MEETING (%) | Votes in ST G (N 51) |
| Abiraterone | 38 | | 38 | 35 | 18 |
| Enzalutamide | 17 | | 17 | 24 | 12 |
| No preferred choice | 45 | | 45 | 37 | 19 |
| Abstain | 0 | | 0 | 4 | 2 |
| Unqualified to answer | 0 | |  | 0 | 0 |

|  | | 4.6 What is your preferred second-line mCRPC treatment option in the majority of men with asymptomatic mCRPC who had progressive disease as best response to first-line Abiraterone or Enzalutamide? | | | |
| --- | --- | --- | --- | --- | --- |
|  | BEIRUT MEETING (%) | | Percentage of eligible votes Beirut | ST GALLEN MEETING (%) | Votes in ST G (N 51) |
| Abiraterone or Enzalutamide  (depending which has already been used) | 41 | | 41 | 14 | 7 |
| Taxane | 56 | | 56 | 70 | 36 |
| No preferred option | 4 | | 4 | 6 | 3 |
| Abstain | 0 | | 0 | 0 | 0 |
| Unqualified to answer | 0 | |  | 0 | 0 ( 2 rad, 3 sipul) |

|  | | 4.7 What is your preferred second-line mCRPC treatment option in the majority of men with symptomatic mCRPC who had progressive disease as best response to first-line Abiraterone or Enzalutamide? | | | |
| --- | --- | --- | --- | --- | --- |
|  | BEIRUT MEETING (%) | | Percentage of eligible votes Beirut | ST GALLEN MEETING (%) | Votes in ST G (N 50) |
| Abiraterone or Enzalutamide  (depending which has already been used) | 14 | | 14 | 0 | 0 |
| Taxane | 86 | | 85 | 96 | 48 |
| No preferred option | 0 | | 0 | 0 | 0 |
| Abstain ( including other treatment option) | 0 | | 0 | 0 | 0 |
| Unqualified to answer | 0 | |  | 0 | 0 (2 radium-223) |

|  | | 4.8 What is your preferred second-line mCRPC treatment option in the majority of men with asymptomatic mCRPC and secondary (acquired) resistance (initial response followed by progression) after use of first-line Abiraterone or Enzalutamide? | | | |
| --- | --- | --- | --- | --- | --- |
|  | BEIRUT MEETING (%) | | Percentage of eligible votes Beirut | ST GALLEN MEETING (%) | Votes in ST G (N 51) |
| Abiraterone or Enzalutamide  (depending which has already been used) | 27 | | 27 | 27 | 14 |
| Taxane | 73 | | 73 | 57 | 29 |
| No preferred option | 0 | | 0 | 2 | 1 |
| Abstain ( including other treatment option) | 0 | | 0 | 0 | 0 |
| Unqualified to answer | 0 | |  | 0 | 0 (5 rad, 2 sipul) |

|  | | 4.9 What is your preferred second-line mCRPC treatment option in the majority of men with symptomatic mCRPC and secondary (acquired) resistance (initial response followed by progression) after use of first-line Abiraterone or Enzalutamide? | | | |
| --- | --- | --- | --- | --- | --- |
|  | BEIRUT MEETING (%) | | Percentage of eligible votes Beirut | ST GALLEN MEETING (%) | Votes in ST G (N 51) |
| Abiraterone or Enzalutamide  (depending which has already been used) | 0 | | 0 | 0 | 0 |
| Taxane | 100 | | 100 | 90 | 46 |
| No preferred option | 0 | | 0 | 2 | 1 |
| Abstain ( including other treatment option) | 0 | | 0 | 0 | 0 |
| Unqualified to answer | 0 | |  | 0 | 0 (4 rad) |

|  | | 4.10 What is your preferred third-line mCRPC treatment option in the majority of men with mCRPC, progressing on or after second-line Docetaxel for mCRPC AND prior treatment with Abiraterone or Enzalutamide? | | | |
| --- | --- | --- | --- | --- | --- |
|  | BEIRUT MEETING (%) | | Percentage of eligible votes Beirut | ST GALLEN MEETING (%) | Votes in ST G (N 51) |
| Abiraterone or Enzalutamide  (depending which has already been used) | 7 | | 7 | 8 | 4 |
| Cabazitaxel | 81 | | 81 | 61 | 31 |
| Platinum-based chemotherapy | 11 | | 11 | 6 | 3 |
| Abstain ( including other treatment option) | 0 | | 0 | 2 | 1 |
| Unqualified to answer | 0 | |  | 0 | 0 (8 rad, 4no pref op) |

**General imaging**

|  | 4.11 All mentioned imaging options are available  What monitoring by imaging do you recommend for the majority of men with metastatic castration-sensitive/naive prostate cancer? | | | | |
| --- | --- | --- | --- | --- | --- |
|  | | BEIRUT MEETING (%) | Percentage of eligible votes Beirut | ST GALLEN MEETING (%) | Votes in ST G (N 51) |
| Baseline imaging and regular monitoring by imaging  every 3-6 months | | 8 | 8 | 31 | 16 |
| Baseline imaging and follow-up imaging at PSA nadir/completion  of 6 cycles of Docetaxel as part of chemo-hormonal therapy and  again at progression (confirmed PSA rise and/or clinical progression) | | 42 | 42 | 51 | 26 |
| Baseline imaging only and monitoring by PSA alone and  imaging at progression | | 50 | 59 | 18 | 9 |
| Abstain | | 0 | 0 | 0 | 0 |
| Unqualified to answer | | 0 |  | 0 | 0 |

|  | | 4.12 What kind of imaging do you recommend for the majority of men with metastatic castration-sensitive/naive prostate cancer? | | | |
| --- | --- | --- | --- | --- | --- |
|  | BEIRUT MEETING (%) | | Percentage of eligible votes Beirut | ST GALLEN MEETING (%) | Votes in ST G (N 51) |
| CT and bone scintigraphy | 31 | | 31 | 73 | 37 |
| CT alone | 4 | | 4 | 0 | 0 |
| Bone scintigraphy alone | 0 | | 0 | 0 | 00 |
| Next generation imaging for prostate cancer | 65 | | 65 | 25 | 13 |
| Abstain | 0 | | 0 | 2 | 1 |
| Unqualified to answer | 0 | |  | 0 | 0 |

|  | | 4.13What monitoring by imaging do you recommend for the majority of men on first-line mCRPC therapy? | | | |
| --- | --- | --- | --- | --- | --- |
|  | BEIRUT MEETING (%) | | Percentage of eligible votes Beirut | ST GALLEN MEETING (%) | Votes in ST G (N 50) |
| Baseline imaging and regular monitoring by imaging every 3-6 months | 20 | | 20 | 54 | 27 |
| Baseline imaging and follow-up imaging at PSA nadir and  again at progression (confirmed PSA rise and/or clinical progression) | 44 | | 44 | 28 | 14 |
| Baseline imaging only and monitoring by PSA alone and  imaging at progression | 36 | | 36 | 16 | 8 |
| Abstain | 0 | | 0 | 2 | 1 |
| Unqualified to answer | 0 | |  | 0 | 0 |

|  | | 4.14 What kind of imaging do you recommend for the majority of men with mCRPC on first-line therapy? | | | |
| --- | --- | --- | --- | --- | --- |
|  | BEIRUT MEETING (%) | | Percentage of eligible votes Beirut | ST GALLEN MEETING (%) | Votes in ST G (N 51) |
| CT and bone scintigraphy | 31 | | 31 | 74 | 38 |
| CT alone | 8 | | 8 | 2 | 1 |
| Bone scintigraphy alone | 0 | | 0 | 0 | 0 |
| Next generation imaging for prostate cancer | 65 | | 65 | 24 | 12 |
| Abstain | 0 | | 0 | 0 | 0 |
| Unqualified to answer | 0 | |  | 0 | 0 |

**Non-APCCC Questions:**

| 4.15 For the purpose of treatment selection in castration-naïve prostate cancer what is the importance of the “high-volume” definition as per CHAARTED compared to the “high-risk” definition as per LATITUDE? | | |
| --- | --- | --- |
|  | BEIRUT MEETING (%) |  |
| “High-volume as per CHAARTED” is **more** important than  “high-risk as per LATITUDE” | 8 |  |
| “High-volume as per CHAARTED” is **less** important than  “high-risk as per LATITUDE” | 15 |  |
| Both definitions are **equally** important | 69 |  |
| Abstain | 8 |  |
| Unqualified to answer | 0 |  |

| 4.16 For the majority of men with metastatic castration-naïve prostate cancer, which treatment do you recommend (provided reimbursement is not an issue)? | | |
| --- | --- | --- |
|  | BEIRUT MEETING (%) |  |
| ADT + 6x Docetaxel | 12 |  |
| ADT + Abiraterone until progression | 44 |  |
| Either of the two options | 44 |  |
| Abstain | 0 |  |
| Unqualified to answer | 0 |  |
